# Controlled human malaria infection by intramuscular and direct venous inoculation of cryopreserved *Plasmodium falciparum* sporozoites in malaria-naïve volunteers: effect of injection volume and dose on infectivity rates

**DOI:** 10.1186/s12936-015-0817-x

**Published:** 2015-08-07

**Authors:** Gloria P Gómez-Pérez, Almudena Legarda, Jose Muñoz, B Kim Lee Sim, María Rosa Ballester, Carlota Dobaño, Gemma Moncunill, Joseph J Campo, Pau Cisteró, Alfons Jimenez, Diana Barrios, Benjamin Mordmüller, Josefina Pardos, Mireia Navarro, Cecilia Justino Zita, Carlos Arlindo Nhamuave, Alberto L García-Basteiro, Ariadna Sanz, Marta Aldea, Anita Manoj, Anusha Gunasekera, Peter F Billingsley, John J Aponte, Eric R James, Caterina Guinovart, Rosa M Antonijoan, Peter G Kremsner, Stephen L Hoffman, Pedro L Alonso

**Affiliations:** ISGlobal, Barcelona Ctr Int Health Res. (CRESIB), Hospital Clínic-Universitat de Barcelona, Barcelona, Spain; Department of Infectious Diseases, Centre of Tropical Medicine and Travel Medicine, Academic Medical Center, University of Amsterdam, Meibergdreef 9, PO Box 22660, 1100 DD Amsterdam, The Netherlands; Sanaria Inc, Rockville, MD USA; Drug Research Centre (CIM), Biomedical Research Institute Sant Pau (IIB Sant Pau), Hospital de la Santa Creu i Sant Pau, Barcelona, Spain; Department of Pharmacology and Therapeutics, Universitat Autònoma de Barcelona (UAB), Bellaterra, Spain; Antigen Discovery, Inc, Irvine, CA USA; Institut für Tropenmedizin, Eberhard Karls Universität Tübingen and German Centre for Infection Research, 72074 Tübingen, Germany; Centro de Investigação em Saúde de Manhiça, Maputo, Mozambique

**Keywords:** Controlled human malaria infection, Malaria, *Plasmodium falciparum*, Cryopreserved sporozoites, Human challenge infection

## Abstract

**Background:**

Controlled human malaria infection (CHMI) by mosquito bite is a powerful tool for evaluation of vaccines and drugs against *Plasmodium falciparum* malaria. However, only a small number of research centres have the facilities required to perform such studies. CHMI by needle and syringe could help to accelerate the development of anti-malaria interventions by enabling centres worldwide to employ CHMI.

**Methods:**

An open-label CHMI study was performed with aseptic, purified, cryopreserved *P. falciparum* sporozoites (PfSPZ Challenge) in 36 malaria naïve volunteers. In part A, the effect of the inoculation volume was assessed: 18 participants were injected intramuscularly (IM) with a dose of 2,500 PfSPZ divided into two injections of 10 µL (n = 6), 50 µL (n = 6) or 250 µL (n = 6), respectively. In part B, the injection volume that resulted in highest infectivity rates in part A (10 µL) was used to formulate IM doses of 25,000 PfSPZ (n = 6) and 75,000 PfSPZ (n = 6) divided into two 10-µL injections. Results from a parallel trial led to the decision to add a positive control group (n = 6), each volunteer receiving 3,200 PfSPZ in a single 500-µL injection by direct venous inoculation (DVI).

**Results:**

Four/six participants in the 10-µL group, 1/6 in the 50-µL group and 2/6 in the 250-µL group developed parasitaemia. Geometric mean (GM) pre-patent periods were 13.9, 14.0 and 15.0 days, respectively. Six/six (100%) participants developed parasitaemia in the 25,000 and 75,000 PfSPZ IM and 3,200 PfSPZ DVI groups. GM pre-patent periods were 12.2, 11.4 and 11.4 days, respectively. Injection of PfSPZ Challenge was well tolerated and safe in all groups.

**Conclusions:**

IM injection of 75,000 PfSPZ and DVI injection of 3,200 PfSPZ resulted in infection rates and pre-patent periods comparable to the bite of five PfSPZ-infected mosquitoes. Remarkably, it required 23.4-fold more PfSPZ administered IM than DVI to achieve the same parasite kinetics. These results allow for translation of CHMI from research to routine use, and inoculation of PfSPZ by IM and DVI regimens.

Trial registration: ClinicalTrials.gov NCT01771848.

**Electronic supplementary material:**

The online version of this article (doi:10.1186/s12936-015-0817-x) contains supplementary material, which is available to authorized users.

## Background

Malaria is one of the oldest and deadliest foes of mankind. Transmission of this parasitic disease continues in 97 countries worldwide, with 198 million cases of malaria and 584,000 deaths estimated in 2013, 90% of them occurring in Africa [1]. In the same year, malaria killed an estimated 437,000 African children under 5 years of age [1]. Moreover, malaria is an infectious disease not only endemic in poor countries but also an important contributor to the vicious cycle of poverty, having a significant negative impact on economic growth and development in these populations [2, 3]. Its eradication is currently on the global health agenda, an ambitious goal that will require the development, integration and implementation of more powerful strategies than the ones currently available, including new anti-malarial drugs and effective vaccines [4]. Nevertheless, as important as the new tools needed, are the methods to test them.

Controlled human malaria infection (CHMI) has facilitated progress in research and development of vaccines and drugs providing the possibility to infect volunteers under controlled conditions [5, 6]. *Plasmodium falciparum* malaria is a pathogen particularly suited to challenge studies for several reasons: it has a relatively short incubation period, a standardized diagnostic laboratory test (thick smear microscopy) is available, strains known to be sensitive to all anti-malarial drugs can be used, and no known long-term sequelae or infectious state arise if properly treated [7]. First generation CHMI were performed with mosquitoes fed on the blood of volunteers who had been infected with *P. falciparum* by inoculation of blood-stage parasites [8]. After the development of in vitro methods to culture *P. falciparum*, a second generation of CHMI was possible in which mosquitoes were fed upon gametocyte-containing blood from cultures of well-characterized *P. falciparum* strains instead of infected human subjects [8, 9]. This second generation approach to CHMI, which was first reported in 1986 [9] has been iteratively standardized over the subsequent decades [8] and recently formally standardized under the umbrella of the World Health Organization (WHO) [10]. It generally involves the administration of *P. falciparum* sporozoites (PfSPZ) by the bites of five PfSPZ-infected *Anopheles stephensi* mosquitoes [8, 10], although exposure to the bites of three aseptically reared *An. stephensi* mosquitoes consistently induces infection [11, 12]. Regardless, current methods of CHMI have facilitated substantial progress in the malaria research field by providing a safe and reproducible method to infect volunteers with *P. falciparum*.

CHMI by the bites of five mosquitoes consistently infects all malaria-naïve volunteers, with rare exceptions, with a geometric mean (GM) pre-patent period (time from exposure to PfSPZ to detection of parasites by microscopy) of less than 12 days (range 7.3–14.5) [6, 8, 11, 13]. Mosquito bite CHMI, although being considered the more natural route of infection, has the limitation of not allowing any calculation of the number of sporozoites (SPZ) inoculated per challenge, therefore leading to a large biological variability in the number of parasites injected per study participant. However, malaria research centres have achieved remarkable experience and standardization performing the second generation of mosquito-administered CHMI since 1986, with more than 1,500 volunteers challenged so far [8, 14, 15]. Nevertheless, these studies are complex in nature and as a result, can be performed only in a few research centres worldwide. With the increasing number of candidate vaccines and drugs in the research and development pipeline, it is necessary to increase accordingly the testing capacity and make CHMI studies broadly accessible, especially in malaria-endemic regions [7].

In the early 1950s, researchers at the National Institutes of Health, USA, were able to isolate *Plasmodium malariae* SPZ, preserve them at temperatures under −70°C for as long as 375 days and administer them to volunteers [16]. Infection rates obtained in trials performed at that time were variable and SPZ preparations highly contaminated [17]. Currently however, it is possible to produce infectious, aseptic, purified, vialed, cryopreserved PfSPZ (PfSPZ Challenge) that can be injected by needle and syringe, a method that could represent the third generation of CHMI. Nevertheless, in order to provide an alternative to the standard mosquito-administered CHMI, PfSPZ Challenge will need to achieve the same results by consistently infecting 100% of volunteers with parasite kinetics similar to mosquito-administered infections.

The present PfSPZ Challenge study was designed based on the results of trials conducted in Nijmegen (The Netherlands) [17], Oxford (UK) [7], and Tübingen (Germany) [18]. These three studies were designed to answer specific questions regarding the dose of cryopreserved PfSPZ, number of injection sites, and route of inoculation needed to achieve results similar to CHMI by mosquito bite regarding infectivity rate (100%) and GM pre-patent period (≤12 days). The first PfSPZ Challenge study performed in The Netherlands showed that injection of PfSPZ Challenge is infectious, safe and well tolerated [17]. This research group tested the intradermal (ID) route with doses of 2,500, 10,000 and 25,000 PfSPZ divided into two 50-µL injections. Infectivity rates and pre-patent periods obtained were not comparable to the bite of five infected mosquitoes. The second trial performed in the UK achieved for the first time a 100% infectivity rate with intramuscular (IM) injection of 25,000 PfSPZ divided into two 50-µL injections, with a GM pre-patent period of 12.7 days. The lack of dose response in this trial opened questions about other factors possibly affecting the efficacy of the inoculation. The third PfSPZ Challenge study performed in Germany tested for the first time the intravenous (IV) route in a dose escalation trial, which assessed 50, 200, 800, and 3,200 PfSPZ administered [18]. As expected from the results in animal models [19], the IV route obtained optimal results with a dose of 3,200 PfSPZ in a single 500-µL injection by IV catheter, resulting in 100% infectivity rate and a GM pre-patent period of 11.2 days. To complete the series of optimization trials of PfSPZ Challenge, the objective of the present study was to assess at which dose and volume of injection does PfSPZ IM administration achieve infection kinetics equivalent to PfSPZ IV inoculation and five mosquito bites. Results of this study have major implications for future CHMI based in IM injection of PfSPZ.

## Methods

### Objectives

The primary objectives of the study were to assess in part A (1) the effect of changing the volume of IM injection; and, in part B (2) increasing the dose of PfSPZ by IM injection using the optimal volume determined in part A, on infectivity rates and pre-patent periods of PfSPZ Challenge. The secondary objective was to assess the safety of PfSPZ Challenge administered in various regimens. Additionally, this study aimed to verify the reproducibility of the results obtained in the PfSPZ Challenge study at the Institut für Tropenmedizin, Universität Tübingen (UKT) in Germany, that achieved 100% infectivity rate with a GM pre-patent period of 11.2 days (range 10.5–12.5) in nine volunteers injected with a dose of 3,200 PfSPZ in a volume of 500 µL by IV catheter.

### Study design and participants

The study was an open-label, non-randomized, controlled study in healthy adults conducted at the ISGlobal-Barcelona Centre for International Health Research (CRESIB), Hospital Clínic-Universitat de Barcelona and at the Drug Research Centre (CIM), Institute of Biomedical Research (IIB), Research Institute of Santa Creu and Sant Pau Hospital, Barcelona, Spain from December 2012 to July 2013. Volunteers 18–45 years of age were invited to participate and screened for eligibility. The main target group for recruitment was students from the Universitat de Barcelona (UB) and their close relatives and friends. Two information sessions (~150 students attended in total) were held at the UB School of Medicine to explain the study and invite subjects to participate. Subsequently, interested students (groups of 4–14) were interviewed by the study physicians to provide more detailed information and answer questions. Additionally, institutional emails (ISGlobal, CRESIB, Hospital Clínic) were sent informing and inviting participation in the study. Eligibility was assessed by medical history, physical examination, standard haematological and biochemical tests, measurement of antibodies to asexual, erythrocytic *P. falciparum* parasites by indirect immunofluorescence test [IIFT, EUROINMUNO Medizinische Labordiagnostika AG; EUROPLUS: *Plasmodium falciparum/Plasmodium vivax* (IgG-IgM)], human immunodeficiency virus (HIV), hepatitis B, and hepatitis C serology, urine pregnancy test, electrocardiogram and risk assessment of cardiovascular disease. Main exclusion criteria were residence in a malaria-endemic region within the previous 6 months, history of receiving an investigational malaria vaccine, positive *P. falciparum* serology, pregnancy, any finding that suggested a chronic condition, or that in the opinion of the study physician, could significantly increase the risk to the volunteer of developing severe malaria infection.

Before any intervention (medical history, screening tests) all volunteers gave written informed consent and answered correctly a questionnaire aimed to test their understanding of the study. They were requested to give three close contact phone numbers and social security numbers, to be able to search for them in case of missing follow-up visits. The study received approval by the ethics committees of Hospital Clínic and Hospital de la Santa Creu i Sant Pau together with the administrative clearance provided by the Health Department of Generalitat de Catalunya. The study followed the principles of the Declaration of Helsinki in its 6th revision (2008) as well as International Conference on Harmonization—Good Clinical Practice (ICH-GCP) guidelines. The study is registered with ClinicalTrials.gov number NCT01771848.

### PfSPZ challenge product

SPZ from *P. falciparum* strain NF54 fully susceptible to chloroquine were purified, and cryopreserved at a specified concentration at Sanaria Inc. facilities as described [7, 15, 17, 20]. PfSPZ Challenge is dispensed into screw-cap vials containing 15,000, 50,000 or 100,000 PfSPZ in 20-µL aliquots and stored in liquid nitrogen vapour phase at −140 to −196°C [20]. Two separate lots of PfSPZ were used in the Barcelona trial: one lot for part A and a second lot for part B, produced in March 2011 and July 2012, respectively. PfSPZ Challenge released for clinical use meets quality control specifications including sterility, purity and potency [20, 21]. The quality control release and stability programme assessed potency and viability using in vitro infection of cultured human hepatocytes (HC-04) and a membrane integrity assay (see Additional file 1: Table S1), respectively [17, 20, 21]. Briefly, 50,000 PfSPZ were added to 40,000 HC-04 (1F9) cells and cultured for 6 days. Late liver stage parasites were detected by staining with a monoclonal antibody against *P. falciparum* merozoite protein 1. Membrane integrity was tested by fluorescence microscopy of PfSPZ following incubation with SYBR green and propidium iodide. Volunteers were inoculated within 30 min after thawing of PfSPZ.

### Inoculation of PfSPZ challenge

**Fig. 1:**
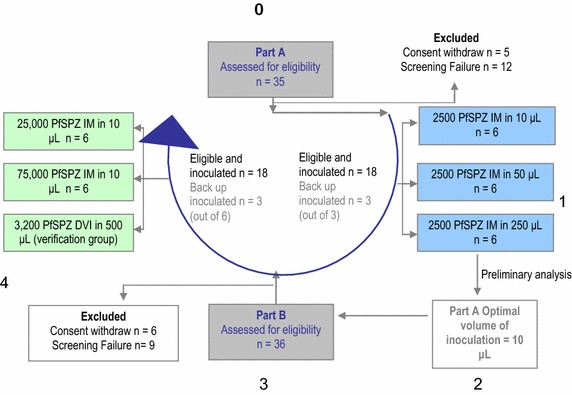
Flow chart of volunteer recruitment. This trial was divided in two parts. In *part A* the impact of volume of inoculation on infectivity rate and pre-patent period was tested by injecting the same dose of *P. falciparum* sporozoites (PfSPZ) in three different injection volumes. In *part B*, the volume that resulted in the highest infectivity rate was used for the formulation of two increased IM doses. A DVI group was included in *part B* to independently corroborate the results of the trial in Tübingen [18]. *IM* intramuscular, *DVI* direct venous inoculation, *back up* refers to the extra volunteers who were enrolled in each part of the study. Group 1: 2,500 PfSPZ in 10 μL; group 2: 2,500 PfSPZ in 50 μL; group 3: 2,500 PfSPZ in 250 μL; group 4: 3,200 PfSPZ in 500 μL; group 5: 25,000 PfSPZ in 10 μL; group 6: 75,000 PfSPZ in 10 μL.

Volunteers were assigned to six different groups (Group 1–6). Three groups were inoculated in part A and three in part B, according to the order of enrolment. Additionally, back-up volunteers were screened and inoculated only if any of the initially assigned volunteers withdrew or did not fulfill the study criteria shortly before the inoculation date (Fig. 1). The inoculation of PfSPZ Challenge took place in January 2013 (part A) and April 2013 (part B) at the Drug Research Centre (CIM), Research Institute of Santa Creu and Sant Pau Hospital, Barcelona. In part A, 18 volunteers received a dose of 2,500 PfSPZ divided in two IM injections (1,250 PfSPZ per injection) in three different volume suspensions: Group 1, 10 µL (n = 6), Group 2, 50 µL (n = 6) and Group 3, 250 µL (n = 6), one injection in each deltoid. In part B, the optimal injection volume determined in part A (10 µL) was used for IM injection of higher doses. Group 5 received 25,000 PfSPZ (n = 6) divided in two separated doses of 12,500 PfSPZ in 10 µL; and Group 6 received 75,000 PfSPZ (n = 6) divided in two separated doses of 37,500 PfSPZ in 10 µL, respectively injected in the deltoid (IM) of each arm. In a third group, Group 4 (n = 6), 3,200 PfSPZ suspended in 500 μL were administered by direct venous inoculation (DVI) into the right arm immediately after venipuncture using a 1 mL syringe with a 25G × 16 mm needle. An IV catheter was inserted in the left arm prior to PfSPZ DVI to serve as emergency access in case of anaphylactic reaction.

### Procedures

All volunteers were observed for at least 1 h after PfSPZ Challenge administration (Day 0) and examined on the following day (Day 1). Participants were provided with a diary where they were instructed to register their daily symptoms and concomitant medication taken, as well as with a card with contact information (telephone numbers of study physicians) and a digital thermometer. Between Day 2 and Day 5 volunteers were contacted daily by telephone. In part A, twice-daily visits and thick blood smears (TBS) were performed from Day 6 until the first positive blood smear or Day 21 was reached. In part B, once-daily visits were performed between Days 6 and 9, followed by twice-daily visits from Day 10 onwards. TBS were performed with blood samples taken by venipuncture in 2-mL EDTA tubes. On the day of first parasite detection by microscopy or Day 21, volunteers started a curative anti-malarial treatment with chloroquine. When chloroquine was contra-indicated as in the case of two volunteers with psoriasis, participants were treated with atovaquone–proguanil. Later follow-up visits of volunteers were performed on Days 35 and 90 after inoculation. Every time blood was taken for TBS, an extra blood sample was drawn in a 2-mL EDTA tube and stored at −80°C for subsequent assessment of sub-microscopic parasitaemia by quantitative real time polymerase reaction (qPCR). qPCR was performed retrospectively once all follow-up visits were completed. Adverse events (AE) and clinical symptoms were reviewed daily and graded according to standard criteria of the US Food and Drug Administration (FDA) guidelines [22] until day of treatment completion and at all follow-up visits thereafter.

### Laboratory method of diagnosis

Successful infection was defined as the appearance of asexual parasites in peripheral blood, detected by TBS microscopy. The pre-patent period was defined as the time between PfSPZ Challenge injection and first positive TBS. Quantitative TBS were prepared as described elsewhere [23]. In brief, 10 μL of blood were spread evenly on a 1 × 2 cm area of a slide, dried in an incubator and stained with Giemsa. Four smears were prepared at each visit. At least two microscopists were required to observe a minimum of two unambiguous parasites in independent readings to declare a slide positive. For each time point the reader performed five passes in one of the four smears (five passes equivalent to assessing 0.5 μL of blood, with some variations depending on the characteristics of the microscope objective lens) when the volunteer was considered asymptomatic by the clinician of the study, and 24 passes when the participant was considered symptomatic. Limit of detection of TBS when reading five passes was ~four parasites per μL. Reading more than five passes improves the detection capacity of the TBS detecting smaller parasite densities. Parasitaemia was estimated by qPCR by methods previously described [24] and performed at the UKT in Germany. Limit of quantification of qPCR was 30 parasites per mL.

### Statistical analysis

**Fig. 2:**
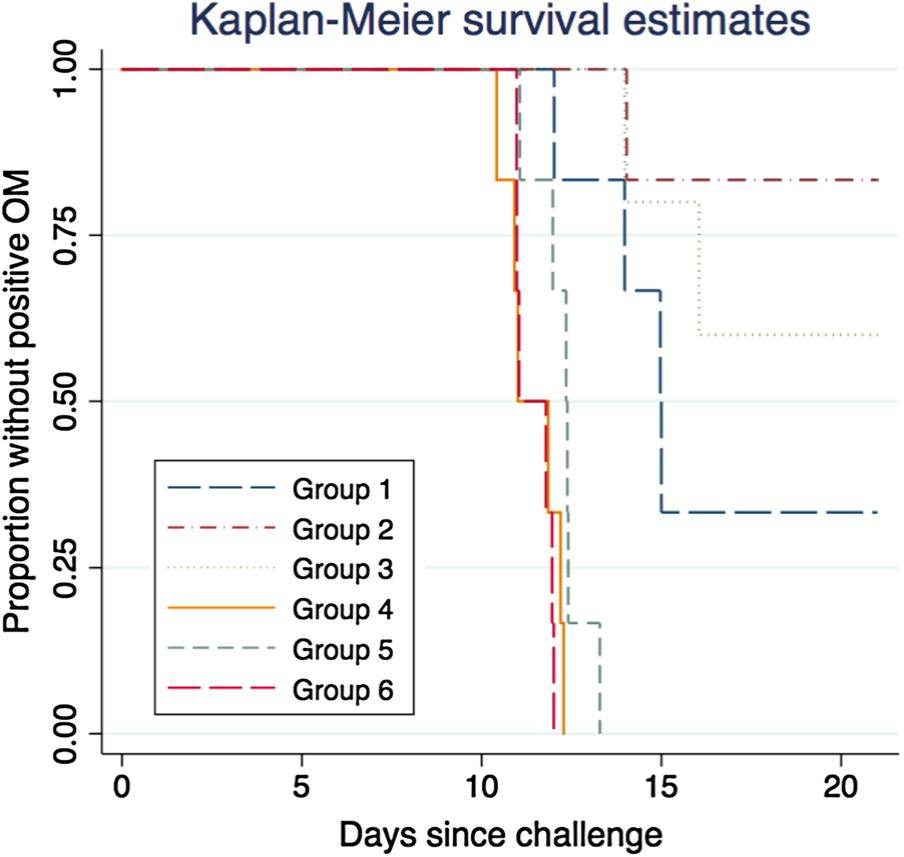
Kaplan–Meier plot of time to infection. *OM* optic microscopy; group 1: 2,500 PfSPZ in 10 μL × 2 IM; group 2: 2,500 PfSPZ in 50 μL × 2 IM. Group 3: 2,500 PfSPZ in 250 μL × 2 IM; group 4: 3,200 PfSPZ in 500 μL × 1 DVI; group 5: 25,000 PfSPZ in 10 μL × 2 IM; group 6: 75,000 PfSPZ in 10 μL × 2 IM; *PfSPZ* *P. falciparum* sporozoites, *IM* intramuscular injection, *DVI* direct venous inoculation.

Data were double-entered using the OpenClinica™ [25] software for clinical data management. The main analysis was done on the intention to treat (ITT) cohort that included all volunteers who underwent CHMI with PfSPZ Challenge. AEs were analysed as pre-erythrocytic phase adverse events or erythrocytic phase adverse events as specified in the correspondent section below. AEs were categorized as related to PfSPZ inoculation, malaria and anti-malarial treatment. The pre-patent period was calculated in days dividing by 24 the number of hours from time of inoculation until time of first positive TBS and summarized by group using geometric means. Times to first positive TBS were visualized using Kaplan–Meier graphs (Fig. 2). All calculations were done with Stata statistical software version 14. Graphs of parasite density by qPCR were done with R version 3.1.0. Graphs of pre-patent periods by groups were done with GraphPad Prism 5.01.

## Results

### Safety

Eligibility criteria was assessed in 71 volunteers from which 36 were enrolled (Fig. 1). Twenty-five of the 36 participants developed *P. falciparum* parasitaemia and all had mild malaria. No volunteer required hospitalization, and all were successfully cured by anti-malarial treatment, no serious AE (SAE) occurred. Twenty-three of the participants who developed malaria had a negative TBS 1 day after onset of treatment. The two volunteers who were still positive by TBS 1 day after onset of treatment became TBS negative 2 days after diagnosis.

### Pre-erythrocytic phase adverse events

The pre-erythrocytic phase was considered the period between injection of PfSPZ Challenge inoculation and Day 5 after injection. Mild local reactogenicity to PfSPZ Challenge was present in one volunteer from Group 3 (2,500 PfSPZ IM in 250 µL) and one volunteer from Group 2 (2,500 PfSPZ IM in 50 µL). The latter scratched the site of injection immediately after the inoculation took place and who had a non-pruritic, painless 4-mm diameter erythematous and edematous area at one of the injection sites that lasted 1 day. This volunteer did not develop parasitaemia. All AEs observed during the pre-erythrocytic phase were transient and the majority were mild (Tables 1, 2). Only two volunteers had headaches, which were of moderate intensity during this period that responded favourably to acetaminophen treatment and lasted a few days. No severe AEs (Grade 3) were observed during the pre-erythrocytic phase and there were no differences in the frequency of AEs between the groups. No SAE occurred.

**Table 1:** Number of AEs or laboratory abnormalities by grade and group in the pre-erythrocytic phase: between day of injection (day 0) and day 5

Severity grade	Challenge group (n = 6 per group)						Total
	Group 1 2,500 PfSPZ 10 µL × 2 IM	Group 2 2,500 PfSPZ 50 µL × 2 IM	Group 3 2,500 PfSPZ 250 µL × 2 IM	Group 4 3,200 PfSPZ 500 µL × 1 DVI	Group 5 25,000 PfSPZ 10 µL × 2 IM	Group 6 75,000 PfSPZ 10 µL × 2 IM	
Grade 1	2	7	5	7	6	2	29
Grade 2	4	1	1	1	2	2	11
Total	6	8	6	8	8	4	40

No grade 3 adverse events were registered during this period.

*PfSPZ* *Plasmodium falciparum* sporozoite, *IM* intramuscular injection, *DVI* direct venous inoculation.

**Table 2:** Type and number of grade 2 adverse events in the pre-erythrocytic phase: between day of injection (day 0) and day 5

Adverse event description	CHMI Groups						Total
	Group 1 2,500 PfSPZ 10 µL × 2 IM	Group 2 2,500 PfSPZ 50 µL × 2 IM	Group 3 2,500 PfSPZ 250 µL × 2 IM	Group 4 3,200 PfSPZ 500 µL × 1 DVI	Group 5 25,000 PfSPZ 10 µL × 2 IM	Group 6 75,000 PfSPZ 10 µL × 2 IM	
Abdominal pain	1	0	0	0	0	0	1
Atopic dermatitis lesions	0	1	0	0	0	0	1
Common cold	0	0	0	0	0	1	1
Diarrhoea	0	0	1	0	0	0	1
Dysmenorrhoea	0	0	0	1	1	1	3
Dyspepsia	1	0	0	0	0	0	1
Headache	1	0	0	0	1	0	2
Muscle spasm	1	0	0	0	0	0	1
Total	4	1	1	1	2	2	11

*PfSPZ* *Plasmodium falciparum* sporozoite, *IM* intramuscular injection, *DVI* direct venous inoculation.

### Erythrocytic phase adverse events

The analysis of the AEs observed during erythrocytic replication was divided into two phases: (1) after day 5 (D5) and before malaria diagnosis or D21 for those who did not develop parasitaemia; and, (2) after treatment, which was on the day of diagnosis for those who developed parasitaemia and on D21 for those who did not develop parasitaemia, through to the completion of follow-up visits on D90.

No Grade 3 AEs were observed during the first erythrocytic phase after D5 and before diagnosis of malaria or D21. The most frequent Grade 2 AE attributed to malaria during this phase was headache (27%), followed by fatigue (7%). Only one volunteer developed a Grade 2 fever (38°C); this occurred one visit before malaria diagnosis. A detailed list of the AEs of moderate intensity (Grade 2) observed during this period is provided in Additional file 2: Table S2.

A total of 377 AEs occurred after the day of malaria diagnosis or D21 and the completion of the follow-up visits (Table 3). The vast majority were mild (Grade 1; n = 235). In addition, 117 moderate (Grade 2) and 25 severe (Grade 3) AEs were observed. The most common Grade 2 AE was again headache (14.5%) follow by fever (9.4%), malaise (6%) and myalgia (5%) (see Additional file 3: Table S3). Two participants presented fever of Grade 2 in between the twice-daily scheduled visits; they were requested to come to the clinic and malaria was diagnosed by TBS. Most of the time symptoms presented together, especially fever and headache. All symptoms were well controlled with acetaminophen. Two volunteers had moderate anaemia 2 days after initiation of malaria treatment, one with haemoglobin of 10.3 g/dL (baseline value 12 g/dL), and the other one with a haematocrit of 32% (baseline value 36%) that persisted until Day 35 (D35) after PfSPZ Challenge inoculation. Iron supplementation was initiated and haemoglobin and haematocrit values normalized until the last follow-up visit 90 days after PfSPZ Challenge (D90). One volunteer, who had normal values of haemoglobin and haematocrit following malaria treatment and at the D35 visit, had moderate anaemia at D90. The volunteer was referred to the tropical medicine department of the Hospital Clínic for further studies. Several participants had transient laboratory abnormalities expected during malaria infections (Table 4). The most common Grade 3 AE was lymphopaenia (32%) followed by fever (28%) and decreased neutrophil count (12%) (see Additional file 4: Table S4). Severe AEs were transient and at frequencies expected following *P. falciparum* infection. No SAE occurred. A summary of the frequency of volunteers that presented Grade 3 AEs in both pre-erythrocytic and erythrocytic stages by inoculation group is shown in Additional file 5: Table S5.

**Table 3:** Number of AEs or laboratory abnormalities by grade and group in the erythrocytic phase: after initiation of treatment of malaria or day 21 until the end of follow up visits on day 90

Severity grade	Challenge group						Total
	(n = 6 for each group)						
	Group 1 2,500 PfSPZ 10 µL × 2 IM	Group 2 2,500 PfSPZ 50 µL × 2 IM	Group 3 2,500 PfSPZ 250 µL × 2 IM	Group 4 3,200 PfSPZ 500 µL × 1 DVI	Group 5 25,000 PfSPZ 10 µL × 2 IM	Group 6 75,000 PfSPZ 10 µL × 2 IM	
No subjects with *P. falciparum* parasitaemia	4	1	2	6	6	6	25
Grade 1	25	21	23	51	53	62	235
Grade 2	18	8	18	28	19	26	117
Grade 3	2	0	4	6	4	9	25
Total	45	29	45	85	76	97	377

*PfSPZ* *Plasmodium falciparum* sporozoite, *IM* intramuscular injection, *DVI* direct venous inoculation.

**Table 4:** Number of volunteers with haematological and biochemical changes at 24 h after initiation of treatment for *Plasmodium falciparum* infection and on days 35 and 90 after injection of PfSPZ Challenge

Parameter	Normal range	Ranges of laboratory abnormalities	Number of volunteers with laboratory abnormalities among the 25 that developed malaria			
			Screening	24 h after first detection of parasitaemia	Day 35 after injection of PfSPZ Challenge	Day 90 after injection of PfSPZ Challenge
Hb level	120–170 g/L	<120 g/L	1 (119 g/L)	5 (lowest value 106 g/L)	5 (lowest value 103 g/L)	3 (lowest value 104 g/L)
WBC count	4.0–11.0 × 10^9^ cells/L	<4.0 × 10^9^ cells/L	2 (lowest value 3.75 × 10^9^ cells/L)	16 (lowest value 2.4 × 10^9^ cells/L)	1 (3.6 × 10^9^ cells/L)	0
Lymphocyte count	0.9–4.5 × 10^9^ cells/L	<0.9 × 10^9^ cells/L	0	16 (lowest value 0.3 × 10^9^ cells/L)	0	0
Platelet count	130–400 × 10^9^ cells/L	<130 × 10^9^ cells/L	0	6 (lowest value 68 × 10^9^ cells/L)	0	0
ALT	5–40 IU/L	≥1.1 ULN	0	6 (highest value 159 IU/L)	0	0
AST	5−40 IU/L	≥1.1 ULN	1 (58 IU/L)	6 (highest value 125 IU/L)	0	1 (44 IU/L)
LDH	<450 IU/L	≥450 IU/L	2 (highest value 515 IU/L)	9 (highest value 652 IU/L)	1 (466 IU/L)	0
Creatinine	0.3–1.3 mg/dL	Any abnormality	0	0	0	0

*HB* haemoglobin, *WBC* white blood cells, *ALT* alanine aminotransferase, *AST* aspartate transaminase, *LDH* lactate dehydrogenase.

Two volunteers presented during clinical malaria with additional symptoms. One 40-years old female, who received 3,200 PfSPZ by DVI, was diagnosed with malaria with a parasitaemia of 1.25 parasites/µL 10 days after inoculation of PfSPZ Challenge. She received chloroquine treatment immediately after, with malaria symptoms lasting 24 h and with a negative TBS 1 day after onset of treatment. Between the third and the fourth dose of chloroquine, (24 and 48 h after the first dose, respectively), she developed anxiety, insomnia, anhedonia and anticipatory fear. As malaria symptoms were absent and the TBS was negative; she was referred to the psychiatry department of the Hospital Clínic where she was diagnosed with unspecified anxiety disorder. She received benzodiazepines for 2 months and was safely managed as an outpatient. No anxiety was reported at D90 visit. Another female volunteer, a 20-years old, UB medical student, who received 75,000 PfSPZ IM, was diagnosed with malaria with a parasitaemia of 3.3 parasites/µL 12 days after injection of PfSPZ Challenge. She presented after the second dose of chloroquine (6 h after onset of treatment) with parasternal chest pain of moderate intensity, described as a burning sensation, accompanied by shortness of breath after moderate effort. Due to the antecedent of a cardiac AE after initiation of treatment for malaria in a CHMI in The Netherlands [26], she was evaluated in the emergency room of the Hospital Clínic shortly after the onset of symptoms. Oxygen saturation was 98% on room air, electrocardiogram (EKG) showed non-specific changes, and haematological and biochemical tests, including cardiac enzymes, were normal, except for D-dimer that was increased with a value of >10,000 ng/mL (normal <500 ng/mL). Increased plasma concentrations of D-dimer have been reported during malaria infections [27], thus once a cardiac event was ruled out, painkillers were administered. The symptoms rapidly and significantly diminished and the volunteer was discharged a few hours later. The following day, symptoms were milder and EKG and CT-pulmonary angiogram were normal. Follow-up EKG and cardiac enzymes were normal during the subsequent 72 h, although she continued to have shortness of breath after moderate exertion. Seven days after the event, a cardiac MRI and haematological and biochemistry tests were normal, including D-dimer values, and she was asymptomatic. During the following visits, the volunteer was asymptomatic with no abnormalities in blood tests. Neither of these two volunteers had history of similar events, and both denied illegal drug consumption.

### Infectivity of PfSPZ challenge

Thirty-six healthy volunteers, 20 women and 16 men between ages 19 and 41, were enrolled out of a group of 71 volunteers who were assessed for eligibility (Fig. 1; Table 5). Demographic characteristics were similar in all groups (Table 5). TBS diagnosis was made promptly, with parasite densities ranging from 0.83 to 56 parasites/µL. In part A, 4/6, 1/6 and 2/6 volunteers developed parasitaemia in the 10, 50 and 250 µL groups, respectively. GM pre-patent periods were 13.9, 14.0 and 15 days, respectively. Since the infection rate was highest in the 10-µL inoculum group, IM dose escalation was done using 10-µL injections.

**Table 5:** Demographic characteristics of the participants

Variable	Group 1 2,500 PfSPZ in 10 µL IM N = 6 Median (Min; max)	Group 2 2,500 PfSPZ in 50 µL IM N = 6 Median (Min; max)	Group 3 2,500 PfSPZ in 250 µL IM N = 6 Median (Min; max)	Group 4 3,200 PfSPZ in 500 µL DVI N = 6 Median (Min; max)	Group 5 25,000 PfSPZ in 10 µL IM N = 6 Median (Min; max)	Group 6 75,000 PfSPZ in 10 µL IM N = 6 Median (Min; max)	Total N = 36 Median (Min; max)
Age (years)	33	26	30	29	22	25	26
	(21; 41)	(21; 32)	(24; 35)	(19; 40)	(20; 25)	(20; 32)	(19; 41)
Weight (Kg)	63	67	80	61	60	54	63
	(58; 86)	(51; 79)	(58; 93)	(54; 73)	(51; 70)	(52; 79)	(51; 93)
Height (m)	1.71	1.71	1.74	1.69	1.7	1.64	1.7
	(1.68; 1.75)	(1.65; 1.86)	(1.63; 1.85)	(1.59; 1.72)	(1.56; 1.75)	(1.60; 1.84)	(1.56; 1.86)
BMI (kg/m2)	22	21.5	26.2	22.8	21.3	20.3	22
	(20.1; 28.1)	(18.0; 25.8)	(21.9; 30.7)	(19.1; 24.8)	(19.9; 22.8)	(19.4; 23.5)	(18.0; 30.7)
Sex							
Female	2 (33%)	3 (50%)	1 (17%)	4 (67%)	5 (83%)	5 (83%)	20 (56%)
Male	4 (67%)	3 (50%)	5 (83%)	2 (33%)	1 (17%)	1 (17%)	16 (44%)

*kg* kilogram, *m* meter, *BMI* body mass index, *Min* minimum value of the range, *Max* maximum value of the range.

In part B, 18/18 (100%) volunteers developed parasitaemia. GM pre-patent periods were 11.4, 12.2 and 11.4 days for Group 4 (3,200 PfSPZ DVI), Group 5 (25,000 PfSPZ IM) and Group 6 (75,000 PfSPZ IM), respectively (Fig. 2; Table 6). There is a significant difference between the pre-patent periods of Group 4 and Group 5 (Kruskal–Wallis test, p = 0.0374), while there is not a statistically significant difference between Group 4 and Group 6 pre-patent periods (Kruskal–Wallis test, p = 1.0000).

**Table 6:** Parasitaemia data by microscopy and qPCR and pre-patent periods by group

	Group 1	Group 2	Group 3	Group 4	Group 5	Group 6
	2,500 PfSPZ 10 µL × 2 IM	2,500 PfSPZ 50 µL × 2 IM	2,500 PfSPZ 250 µL × 2 IM	3,200 PfSPZ 500 µL × 1 DVI	25,000 PfSPZ 10 µL × 2 IM	75,000 PfSPZ 10 µL × 2 IM
	N = 6	N = 6	N = 6	N = 6	N = 6	N = 6
Number of volunteers who became thick blood smear positive (TBS+)	4	1	2	6	6	6
Listing of times to TBS+ (days)	14.0, 15.0, 15.0, 12.0	14.0	16.0, 14.0	11.0, 10.4, 12.3, 10.9, 11.9, 12.2	12.4, 12.4, 13.3, 12.4, 11.1, 12.0	11.8, 11.0, 11.0, 11.0, 12.0, 12.0
Geometric mean time to TBS+ (days)	13.9	14.0	15.0	11.4	12.2	11.4
Listing of parasite density at time of TBS+ (parasites/µL blood)	10.0, 48.0, 6.0, 5.0	34.0	10.0, 27.0	5.0, 1.3, 10.0, 8.0, 12.0, 14.0	4.0, 10.0, 5.8, 10.0, 3.8, 3.3	56.0, 0.8, 0.8, 6.0, 3.3, 6.6
Geometric mean parasite density at time of TBS+ (parasites/µL blood)	11.0	34.0	16.4	6.6	5.5	4.1
Listing of times to qPCR+ (days)	11.0, 11.3, 11.4, 10.0	9.1	11.0, 8.0	6.9, 9.1, 8.0, 8.0, 9.0, 8.9	10.1, 8.0 9.1, 7.0 7.0, 7.1	6.9, 7.0, 7.0, 7.0 7.0, 6.0
Geometric mean of time to qPCR+ (days)	10.9	9.1	9.3	8.3	7.9	6.8

**Fig. 3:**
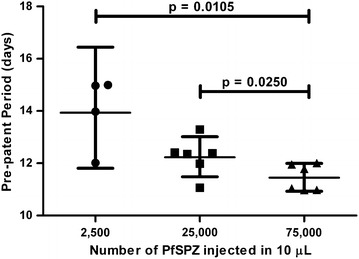
Relationship between dose of PfSPZ Challenge and pre-patent period. Geometric means (*horizontal bars*) and 95% confidence intervals (*vertical bars*) for pre-patent periods. Individual pre-patent periods are shown.

As the PfSPZ dose was increased from 2,500 to 25,000 to 75,000 PfSPZ in a constant 10-μL volume administered IM, the pre-patent period decreased: Group 1 (2,500 PfSPZ in 10 µL IM) had an infectivity rate of 67% and a GM pre-patent period of 13.9 days; Group 5 (25,000 PfSPZ in 10 µL IM) had an infectivity rate of 100% and a GM pre-patent period of 12.2 days; and Group 6 (75,000 PfSPZ in 10 µL IM) had an infectivity rate of 100% and a GM pre-patent period of 11.4 days. Pre-patent periods compared between these three groups were significantly different (Kruskal–Wallis test, p = 0.0080) which was attributed to the reduction in the pre-patent period in the 75,000 PfSPZ dose group compared to that in the 2,500-dose group (Mann–Whitney test, p = 0.0105) or the 25,000-dose group (Mann–Whitney test, p = 0.0250) (Fig. 3). Additional file 6: Figure S1 shows parasite density by qPCR from Groups 1, 5 and 6 to visualize the changes in parasite kinetics as the PfSPZ dose increases.

The results of the UKT trial using IV injection were successfully replicated using DVI. In the present study, volunteers in the 3,200 PfSPZ Challenge DVI group received a different lot than in the UKT trial of PfSPZ Challenge administered by a different research team with a 100% infectivity rate and a GM pre-patent period of 11.4 days. Comparable results were obtained with the 75,000 PfSPZ Challenge IM group in which all volunteers were infected with a GM pre-patent period of 11.4 days. All volunteers receiving 25,000 PfSPZ IM were infected but the GM pre-patent period was extended to 12.2 days.

### Parasite kinetics measured by qPCR

**Fig. 4:**
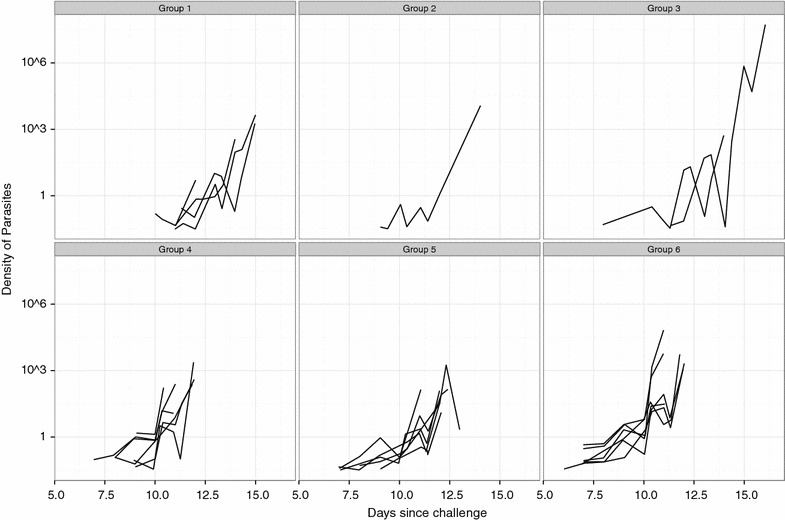
Parasite kinetics measured by qPCR per group. Density units are parasites/mL; Limit of quantification of qPCR was 30 parasites per mL. Group 1: 2,500 PfSPZ in 10 μL × 2 IM; group 2: 2,500 PfSPZ in 50 μL × 2 IM. Group 3: 2,500 PfSPZ in 250 μL × 2 IM; group 4: 3,200 PfSPZ in 500 μL × 1 DVI; group 5: 25,000 PfSPZ in 10 μL × 2 IM; group 6: 75,000 PfSPZ in 10 μL × 2 IM; *PfSPZ* *Plasmodium falciparum* sporozoites, *IM* intramuscular injection, *DVI* direct venous inoculation.

All positive TBS results were confirmed by qPCR except for the results of one volunteer from part A who received 2,500 PfSPZ IM in two 250-µL injections. The TBS from this volunteer was read again by two microscopists after the qPCR results and no parasites were found, thus confirming the false positive TBS result. The pre-patent period of this volunteer was not included in the analysis of the GM pre-patent period of the corresponding group, and this result did not affect the outcome of the preliminary analysis in part A regarding the optimum volume of inoculation. None of the TBS negative volunteers reached qPCR positivity threshold. Figure 4 and Additional file 7: Figure S2 (in colours) plots the qPCR results per group of inoculation. On average, qPCR detected parasites 4.0 days (range 1.4–6.0) before microscopy (see Additional file 8: Table S6).

## Discussion

PfSPZ Challenge administered by IM and DVI injection is safe and well tolerated. The clinical observations here described are in agreement with previously reported clinical data from both mosquito- and needle and syringe-administered CHMI trials [7, 8, 13, 17, 28]. Most of the AEs (51%) were attributed (possibly, probably or definitely related) to malaria and few to PfSPZ inoculation (2%). TBS diagnosis was made promptly and volunteers were treated immediately. All participants responded favourably to a curative dose of chloroquine or atovaquone–proguanil. Both drugs were effective. Nevertheless, there were two clinical cases that caused concern. One subject developed significant anxiety and another non-cardiac, parasternal chest pain and excertional dyspnea after initiation of chloroquine treatment for *P. falciparum* parasitaemia. Review of the screening procedures and the clinical history of the volunteers indicated that there was nothing that would have suggested that these two volunteers were at increased risk for a reaction to treatment of malaria with chloroquine. However, the volunteer with anxiety and her family indicated she had a tendency to be anxious. Nonetheless, it is considered possible that chloroquine treatment was the cause of the anxiety symptoms described above, since such AEs have been reported in relation to this anti-malarial drug [29]. There is not certainty about what caused the non-cardiac parasternal chest pain and exertional dyspnoea. Malaria is sometimes associated with chest pain, and it has been reported that severe malaria with high parasitaemia, which this subject did not have, can mimic acute myocardial infarction [30].

First it was assessed whether increasing the volume of inoculation of 2,500 PfSPZ IM from 10 to 50 to 250 µL changed the infectivity rate and/or pre-patent period. The sample was small and the differences between groups not significant, but among the volunteers receiving 2,500 PfSPZ, the highest infection rate (4/6) and the shortest pre-patent period (13.9 days) occurred in those volunteers administered 2,500 PfSPZ in 10 μL. As the dose was increased from 2,500 to 25,000 to 75,000 PfSPZ in a constant 10-μL volume IM, the infectivity rate increased and the pre-patent period decreased: Group 1 (2,500 PfSPZ in 10 µL IM) had an infectivity rate of 67% and a GM pre-patent period of 13.9 days (range 12.0–15.0); Group 5 (25,000 PfSPZ in 10 µL IM) had an infectivity rate of 100% and a GM pre-patent period of 12.2 days (range 11.1–13.3); and Group 6 (75,000 PfSPZ in 10 µL IM) had an infectivity rate of 100% and a GM pre-patent period of 11.4 days (range 11.0–12.0).

Additionally, the findings of the UKT trial were corroborated [18]. In a different setting, with a different research team, lot of parasites and characteristics of volunteers, there were obtained almost identical results to those in the trial in Germany. The group (N = 6) that received 3,200 PfSPZ by DVI had a 100% infectivity with a GM pre-patent period of 11.4 days, and the group (N = 9) in Germany that received 3,200 PfSPZ IV had a 100% infectivity rate with a GM pre-patent period of 11.2 days [18].

Volunteers injected with 3,200 PfSPZ by DVI or 75,000 PfSPZ in 10 µL IM had infectivity rates and same GM pre-patent periods (11.4 days) comparable to CHMI by exposure to five PfSPZ-infected mosquito bites. This is the first clear comparison in humans between the infection rates after DVI and IM injection. It required 23.4-fold more PfSPZ administered IM than DVI to achieve the same infection rate and pre-patent period. Murine studies predicted that IV/DVI would be more efficient than IM injection, but not to this degree [19]. Intradermal (ID) injection is not more efficient than IM, and in fact may be less efficient [7, 15, 17]. It will be a significant challenge to increase the efficiency of IM and ID injection to make them more comparable to IV/DVI injection. Therefore, DVI of PfSPZ Challenge will be the best way, compared to the IM and ID inoculation, to progress CHMI studies to investigate anti-malarials, diagnostic tools, vaccine-induced immunity targeting liver stages and erythrocytic stages, and will be comparable to CHMI administered by exposure to five PfSPZ-infected mosquito bites. It is expected that DVI with PfSPZ Challenge will also provide comparable results for vaccine-induced immunity targeting PfSPZ. However, it has been argued that antibodies in the skin may play a role in such immunity, and bypassing the skin with DVI injection of PfSPZ may underestimate the effect of such antibodies [31]. This will need to be assessed in a clinical trial.

## Conclusions

Administration of two different quantities of PfSPZ Challenge by two different routes of administration (3,200 PfSPZ by DVI and 75,000 PfSPZ in 10 µL by IM injection), were safe and comparable to mosquito-administered CHMI. In consequence these inoculation regimens represent an alternative tool to mosquito-administered CHMI for researchers worldwide, especially in malaria-endemic countries where vaccine and drug efficacy trials in semi-immune and immune individuals are greatly needed. Such studies in endemic countries will also provide a unique opportunity to study the impact of adaptive immunity and innate phenotypes (e.g., haemoglobinopathies) on infection rates, pre-patent periods and clinical manifestations of malaria in different populations with different levels of *P. falciparum* exposure, studies that may be useful for the development and fielding of future malaria vaccines.

## Additional files

Additional file 1:
**In vitro infectivity to a hepatocyte line (HC-04) (potency) and sporozoite membrane integrity (viability) of the two lots of PfSPZ Challenge**. This table provides data about potency and viability of PfSPZ used for preparation of IM and IV injections for the CHMI in Barcelona in both Part A and Part B. Additional file 2:
**Type and number of grade 2 adverse events during the erythrocytic phase - between Day 6 after injection of PfSPZ Challenge and the day of malaria diagnosis or Day 21.** This table provides a list of AEs observed between Day 6 after injection of PfSPZ Challenge and the day of malaria diagnosis or Day 21, and their frequency in the different inoculation groups. Additional file 3:
**Number of grade 2 adverse events or laboratory abnormalities in the erythrocytic phase - after initiation of treatment of malaria or Day 21 until the end of follow up visits on Day 90.** This table provides a list of AEs observed after initiation of treatment of malaria or Day 21 until the end of follow up visits on Day 90, and their frequency in the different inoculation groups. Additional file 4:
**Type and number of grade 3 adverse events or laboratory abnormalities in the erythrocytic phase – after initiation of treatment of malaria or Day 21 until the end of follow up visits on Day 90.** This table provides a list of grade 3 AEs or laboratory abnormalities observed after initiation of treatment of malaria or Day 21 until the end of follow up visits on Day 90, in the different inoculation groups. Additional file 5:
**Summary of the frequency of grade 3 adverse events by inoculation group.** This table provides about the frequency of grade 3 AEs and their relateness to PfSPZ injection, malaria or malaria treatment in the different inoculation groups. Additional file 6:
**Parasite kinetics measured by qPCR from Groups 1 (2,500 10μL IM), 5 (25,000 10μL IM) and 6 (75,000 10μL IM).** This figure shows parasite densities measured by qPCR, plotted in a single chart and different colors per group, from Groups inoculated by IM injection with increasing doses of 2,500 PfSPZ, 25,000 PfSPZ and 75,000 PfSPZ in a volume of 10μL. The higher the PfSPZ dose inoculated, the sooner sub-microscopic parasite densities are detected. Density units are in parasites/mL. Limit of quantification of qPCR was 30 parasites per mL. Additional file 7:
**Parasite kinetics measured by qPCR from all Groups.** This figure shows parasite densities measured by qPCR, plotted in a single chart and different colors, from all inoculation Groups. Additional file 8:
**Pre-patent periods and time to positivity by qPCR from all Groups.** This table provides data of the pre-patent periods (days) and time to positivity by qPCR (days) per volunteer from all Groups.

## Abbreviations

AE: adverse event
CHMI: controlled human malaria infection
CRESIB: Barcelona Centre for International Health Research
CHMI: controlled human malaria infection
D35: day 35 after challenge
D90: day 90 after challenge
DVI: direct venous inoculation
EKG: electrocardiogram
GM: geometric mean
ID: intradermic
IFA: immunofluorescence assay
IM: intramuscular
ITT: intention to treat
IV: intravenous
PfSPZ: *Plasmodium falciparum* sporozoites
PfSPZ Challenge: aseptic, purified, vialed, cryopreserved *Plasmodium falciparum* sporozoites
pre-patent period: the time between PfSPZ Challenge injection and first positive TBS
qPCR: quantitative polymerase chain reaction
SPZ: sporozoite(s)
TBS: thick blood smear
UB: University of Barcelona
UKT: Institut für Tropenmedizin, Eberhard Karls Universität Tübingen and German Centre for Infection Research
