# Supplementary material for: Controlled human malaria infection by intramuscular and direct venous inoculation of cryopreserved Plasmodium falciparum sporozoites in malaria-naïve volunteers: effect of injection volume and dose on infectivity rates
Source: Malar J. 2015 Aug 7;14:306. doi: 10.1186/s12936-015-0817-x (PMC4527105; doi:10.1186/s12936-015-0817-x)
Supplement: Additional file 1: — In vitro infectivity to a hepatocyte line (HC-04) (potency) and sporozoite membrane integrity (viability) of the two lots of PfSPZ Challenge. This table provides data about potency and viability of PfSPZ used for preparation of IM and IV injections for the CHMI in Barcelona in both Part A and Part B. [file 12936_2015_817_MOESM1_ESM.doc]

**Additional file 1**. ***In vitro* infectivity to a hepatocyte line (HC-04) (potency) and**

**sporozoite membrane integrity (viability) of the two lots of PfSPZ Challenge.**

| **Part A -** **Lot 031611-02^*^** | | |
| --- | --- | --- |
| Time Point | Potency  (number of parasites expressing  PfMSP-1/well at 6 days) | % Viability  (sporozoite membrane integrity assay) |
| Fresh | 32.7 ± 1.5 parasites | 98.2% |
| Release | 29.3 ± 3.1 parasites | 87.4% ± 5.9% |
| 3 Month | 27.3 ± 0.6 parasites | 84.6% ± 1.9% |
| 6 Month | 26.7 ± 1.5 parasites | 83.6% ± 5.5% |
| 9 Month | 26.3 ± 2.5 parasites | 86.3% ± 6.5% |
| 12 Month | 27.3 ± 0.6 parasites | 86.2% ± 1.3% |
| 18 Month | 24.0 ± 1.7 parasites | 81.7% ± 2.6% |
| Post-last clinical dose (24 Month) | 24.0 ± 3.6 parasites | 82.8% ± 2.87% |
| **Part B - Lot 071112-02^¥^** | | |
| Time Point | Potency  (number of parasites expressing  PfMSP-1/well at 6 days) | % Viability  (sporozoite membrane integrity assay) |
| Fresh | 28.3 ± 1.5 parasites | 95.5% |
| Release | 25.3 ± 1.5 parasites | 89% ± 2.2% |
| 3 Month | 21.7 ± 1.5 parasites | 85% ± 3.3% |
| 6 Month | 25.0 ± 5.3 parasites | 86% ± 4.8% |
| Post-last clinical dose  (9 Month) | 19.0 ± 1.0 parasites | 85% ± 4.4% |

*Lot 031611-02 for part A was manufactured in March 2011.

^¥^Lot 071112-02 for part B was manufactured in July 2012.
