# Supplementary material for: Controlled human malaria infection by intramuscular and direct venous inoculation of cryopreserved Plasmodium falciparum sporozoites in malaria-naïve volunteers: effect of injection volume and dose on infectivity rates
Source: Malar J. 2015 Aug 7;14:306. doi: 10.1186/s12936-015-0817-x (PMC4527105; doi:10.1186/s12936-015-0817-x)
Supplement: Additional file 2: — Type and number of grade 2 adverse events during the erythrocytic phase - between Day 6 after injection of PfSPZ Challenge and the day of malaria diagnosis or Day 21. This table provides a list of AEs observed between Day 6 after injection of PfSPZ Challenge and the day of malaria diagnosis or Day 21, and their frequency in the different inoculation groups. [file 12936_2015_817_MOESM2_ESM.docx]

**Additional file 2. Type and number of grade 2 adverse events during the erythrocytic phase - between Day 6 after injection of PfSPZ Challenge and the day of malaria diagnosis or Day 21.**

| Adverse Event Description | CHMI Group | | | | | | Total |
| --- | --- | --- | --- | --- | --- | --- | --- |
|  | Group 1  2,500 PfSPZ 10 µL x 2 IM | Group 2  2,500 PfSPZ 50 µL x 2 IM | Group 3  2,500 PfSPZ 250 µL x 2 IM | Group 4  3,200 PfSPZ 500 µL x 1 DVI | Group 5  25,000 PfSPZ 10 µL x 2 IM | Group 6  75,000 PfSPZ 10 µL x 2 IM |  |
| Allergic rhinitis | 0 | 0 | 0 | 0 | 1 | 0 | 1 |
| Anxiety | 0 | 0 | 1 | 0 | 0 | 0 | 1 |
| Back pain | 0 | 0 | 2 | 0 | 0 | 0 | 2 |
| Chills | 0 | 0 | 0 | 0 | 0 | 2 | 2 |
| Common cold | 3 | 1 | 0 | 0 | 1 | 0 | 5 |
| Diarrhea | 0 | 0 | 1 | 0 | 0 | 0 | 1 |
| Dizziness | 0 | 0 | 0 | 1 | 0 | 0 | 1 |
| Dry eye | 1 | 0 | 0 | 0 | 0 | 0 | 1 |
| Dysmenorrhea | 0 | 1 | 0 | 2 | 0 | 0 | 3 |
| Dyspepsia | 0 | 0 | 1 | 2 | 0 | 0 | 3 |
| Fatigue | 1 | 1 | 0 | 1 | 0 | 1 | 4 |
| Fever | 0 | 0 | 0 | 0 | 1 | 0 | 1 |
| Generalized muscle weakness | 0 | 0 | 0 | 1 | 0 | 0 | 1 |
| Headache | 2 | 3 | 5 | 0 | 3 | 2 | 15 |
| Insomnia | 0 | 0 | 1 | 0 | 0 | 0 | 1 |
| Malaise | 0 | 0 | 0 | 0 | 0 | 1 | 1 |
| Muscle spasm | 0 | 0 | 0 | 0 | 0 | 1 | 1 |
| Myalgia | 0 | 0 | 0 | 1 | 0 | 0 | 1 |
| Nausea | 0 | 1 | 0 | 0 | 0 | 0 | 1 |
| Ovulation pain | 0 | 0 | 1 | 0 | 0 | 0 | 1 |
| Pain at inoculation site | 1 | 0 | 0 | 0 | 0 | 0 | 1 |
| Tarsalgia | 0 | 0 | 1 | 0 | 0 | 0 | 1 |
| Psoriatic lesions | 0 | 0 | 1 | 0 | 0 | 0 | 1 |
| Seborrhoeic dermatitis | 0 | 0 | 1 | 0 | 0 | 0 | 1 |
| Sore throat | 0 | 0 | 2 | 0 | 0 | 0 | 2 |
| Sweating | 1 | 0 | 0 | 0 | 0 | 0 | 1 |
| Toothache | 0 | 1 | 0 | 0 | 0 | 0 | 1 |
| Warty lesion on sole of left foot | 0 | 0 | 0 | 1 | 0 | 0 | 1 |
| Total | 9 | 8 | 17 | 9 | 6 | 7 | 56 |

PfSPZ: *Plasmodium falciparum* sporozoite; IM: intramuscular injection; DVI: direct venous inoculation.
