# Supplementary material for: Controlled human malaria infection by intramuscular and direct venous inoculation of cryopreserved Plasmodium falciparum sporozoites in malaria-naïve volunteers: effect of injection volume and dose on infectivity rates
Source: Malar J. 2015 Aug 7;14:306. doi: 10.1186/s12936-015-0817-x (PMC4527105; doi:10.1186/s12936-015-0817-x)
Supplement: Additional file 3: — Number of grade 2 adverse events or laboratory abnormalities in the erythrocytic phase - after initiation of treatment of malaria or Day 21 until the end of follow up visits on Day 90. This table provides a list of AEs observed after initiation of treatment of malaria or Day 21 until the end of follow up visits on Day 90, and their frequency in the different inoculation groups. [file 12936_2015_817_MOESM3_ESM.docx]

**Additional file 3. Number of grade 2 adverse events or laboratory abnormalities in the erythrocytic phase -** **after initiation of treatment of malaria or Day 21 until the end of follow up visits on Day 90.**

| Adverse Event Description | CHMI Group | | | | | | Total |
| --- | --- | --- | --- | --- | --- | --- | --- |
|  | Group 1  2,500 PfSPZ 10 µL x 2 IM | Group 2  2,500 PfSPZ 50 µL x 2 IM | Group 3  2,500 PfSPZ 250 µL x 2 IM | Group 4  3,200 PfSPZ 500 µL x 1 DVI | Group 5  25,000 PfSPZ 10 µL x 2 IM | Group 6  75,000 PfSPZ 10 µL x 2 IM |  |
| Abdominal pain | 1 | 0 | 0 | 0 | 0 | 0 | 1 |
| Alanine aminotransferase increased | 0 | 0 | 1 | 2 | 0 | 0 | 3 |
| Allergic rhinitis | 0 | 0 | 0 | 0 | 1 | 0 | 1 |
| Anemia | 0 | 0 | 0 | 0 | 2 | 1 | 3 |
| Anxiety | 0 | 0 | 1 | 2 | 0 | 0 | 3 |
| Aspartate aminostransferase increased | 0 | 0 | 1 | 1 | 0 | 0 | 2 |
| Atopic dermatitis lesions | 0 | 1 | 0 | 0 | 0 | 0 | 1 |
| Back pain | 1 | 0 | 1 | 0 | 0 | 0 | 2 |
| Bruising | 0 | 0 | 0 | 1 | 0 | 0 | 1 |
| C Reactive protein increased | 0 | 0 | 0 | 1 | 0 | 3 | 4 |
| Chest pain (anxiety) | 0 | 0 | 0 | 0 | 0 | 1 | 1 |
| Chills | 2 | 0 | 0 | 0 | 1 | 0 | 3 |
| Common cold | 1 | 0 | 1 | 1 | 0 | 0 | 3 |
| Diarrhea | 0 | 0 | 1 | 0 | 0 | 0 | 1 |
| Dizziness | 1 | 2 | 0 | 0 | 0 | 0 | 3 |
| Dyspepsia | 0 | 0 | 1 | 0 | 0 | 0 | 1 |
| Dyspnea | 0 | 0 | 0 | 0 | 0 | 1 | 1 |
| Fatigue | 1 | 0 | 0 | 0 | 1 | 1 | 3 |
| Fever | 0 | 0 | 2 | 4 | 3 | 2 | 11 |
| Flu like symptoms | 0 | 2 | 0 | 0 | 0 | 0 | 2 |
| GGT increased | 0 | 0 | 0 | 0 | 0 | 1 | 1 |
| Headache | 5 | 2 | 2 | 3 | 4 | 1 | 17 |
| Hidradenitis | 0 | 0 | 1 | 0 | 0 | 0 | 1 |
| Hypokalemia | 0 | 0 | 0 | 1 | 0 | 2 | 3 |
| Insomnia | 0 | 0 | 1 | 1 | 0 | 0 | 2 |
| LDH increased | 0 | 0 | 0 | 0 | 1 | 3 | 4 |
| Lymphocyte count decreased | 0 | 0 | 0 | 0 | 0 | 4 | 4 |
| Malaise | 2 | 0 | 1 | 1 | 2 | 1 | 7 |
| Muscle spasm | 0 | 0 | 1 | 0 | 0 | 0 | 1 |
| Myalgia | 0 | 0 | 1 | 4 | 0 | 1 | 6 |
| Nausea | 1 | 0 | 0 | 2 | 0 | 0 | 3 |
| Neutrophil count decreased | 0 | 0 | 0 | 1 | 1 | 1 | 3 |
| Pain at site of DVI | 0 | 1 | 0 | 0 | 0 | 0 | 1 |
| Platelet count decreased | 0 | 0 | 0 | 0 | 1 | 0 | 1 |
| Psoriatic lesions | 0 | 0 | 1 | 0 | 0 | 0 | 1 |
| Respiratory upper tract infection | 1 | 0 | 0 | 0 | 0 | 1 | 2 |
| Somnolence | 1 | 0 | 0 | 0 | 0 | 0 | 1 |
| Stye | 0 | 0 | 0 | 1 | 0 | 0 | 1 |
| Tachycardia | 1 | 0 | 0 | 0 | 2 | 0 | 3 |
| Tendinitis in knees and wrists | 0 | 0 | 1 | 0 | 0 | 0 | 1 |
| Vomiting | 0 | 0 | 0 | 2 | 0 | 0 | 2 |
| White blood cell count decreased | 0 | 0 | 0 | 0 | 0 | 2 | 2 |
| Total | 18 | 8 | 18 | 28 | 19 | 26 | 117 |

PfSPZ: *Plasmodium falciparum* sporozoite; IM: intramuscular injection; DVI: direct venous inoculation; GGT: gamma-glutamyltransferase; LDH: lactate dehydrogenase.
