# Supplementary material for: Controlled human malaria infection by intramuscular and direct venous inoculation of cryopreserved Plasmodium falciparum sporozoites in malaria-naïve volunteers: effect of injection volume and dose on infectivity rates
Source: Malar J. 2015 Aug 7;14:306. doi: 10.1186/s12936-015-0817-x (PMC4527105; doi:10.1186/s12936-015-0817-x)
Supplement: Additional file 4: — Type and number of grade 3 adverse events or laboratory abnormalities in the erythrocytic phase – after initiation of treatment of malaria or Day 21 until the end of follow up visits on Day 90. This table provides a list of grade 3 AEs or laboratory abnormalities observed after initiation of treatment of malaria or Day 21 until the end of follow up visits on Day 90, in the different inoculation groups. [file 12936_2015_817_MOESM4_ESM.docx]

**Additional file 4. Type and number of grade 3 adverse events or laboratory abnormalities in the erythrocytic phase** – **after initiation of treatment of malaria or Day 21 until the end of follow up visits on Day 90.**

| **Adverse Event Description** | **CHMI Group** | | | | | **Total** |
| --- | --- | --- | --- | --- | --- | --- |
|  | Group 1  2,500 PfSPZ 10 µL x 2 IM | Group 3  2,500 PfSPZ 250 µL x 2 IM | Group 4  3,200 PfSPZ 500 µL x 1 DVI | Group 5  25,000 PfSPZ 10 µL x 2 IM | Group 6  75,000 PfSPZ 10 µL x 2 IM |  |
| C Reactive protein increased | 0 | 0 | 1 | 0 | 0 | 1 |
| Chills | 0 | 1 | 0 | 0 | 0 | 1 |
| D-dimer increased | 0 | 0 | 0 | 0 | 1 | 1 |
| Fatigue | 0 | 1 | 0 | 0 | 0 | 1 |
| Fever | 1 | 0 | 2 | 1 | 3 | 7 |
| Headache | 0 | 1 | 0 | 0 | 0 | 1 |
| Hypokalemia | 0 | 0 | 0 | 1 | 0 | 1 |
| Lymphocyte count decreased | 1 | 0 | 3 | 1 | 3 | 8 |
| Myalgia | 0 | 1 | 0 | 0 | 0 | 1 |
| Neutrophil count decreased | 0 | 0 | 0 | 1 | 2 | 3 |
| Total | 2 | 4 | 6 | 4 | 9 | 25 |

PfSPZ: *Plasmodium falciparum* sporozoite; IM: intramuscular injection; DVI: direct venous inoculation; Group 2 did not have grade 3 AEs or laboratory abnormalities..
