# Supplementary material for: Controlled human malaria infection by intramuscular and direct venous inoculation of cryopreserved Plasmodium falciparum sporozoites in malaria-naïve volunteers: effect of injection volume and dose on infectivity rates
Source: Malar J. 2015 Aug 7;14:306. doi: 10.1186/s12936-015-0817-x (PMC4527105; doi:10.1186/s12936-015-0817-x)
Supplement: Additional file 5: — Summary of the frequency of grade 3 adverse events by inoculation group. This table provides about the frequency of grade 3 AEs and their relateness to PfSPZ injection, malaria or malaria treatment in the different inoculation groups. [file 12936_2015_817_MOESM5_ESM.doc]

### Additional file 5. Summary of the frequency of grade 3 adverse events by inoculation group.

| **Variable** | | **Group 1** 2,500 PfSPZ 10 µL x 2 IM | **Group 2**  2,500 PfSPZ 50 µL x 2 IM | **Group 3**  2,500 PfSPZ 250 µL x 2 IM | **Group 4**  3,200 PfSPZ 500 µL x 1 DVI | **Group 5**  25,000 PfSPZ 10 µL x 2 IM | **Group 6**  75,000 PfSPZ 10 µL x 2 IM |
| --- | --- | --- | --- | --- | --- | --- | --- |
| **Any AE** | None | 5 (83.3%) | 6 (100.0%) | 5 (83.3%) | 1 (16.7%) | 3 (50.0%) | 1 (16.7%) |
|  | At least one | 1 (16.7%) | 0 ( 0.0%) | 1 (16.7%) | 5 (83.3%) | 3 (50.0%) | 5 (83.3%) |
| **AE related to challenge** | None | 6 (100.0%) | 6 (100.0%) | 6 (100.0%) | 6 (100.0%) | 6 (100.0%) | 6 (100.0%) |
| **AE related to malaria** | None | 5 (83.3%) | 6 (100.0%) | 5 (83.3%) | 1 (16.7%) | 3 (50.0%) | 1 (16.7%) |
|  | At least one | 1 (16.7%) | 0 ( 0.0%) | 1 (16.7%) | 5 (83.3%) | 3 (50.0%) | 5 (83.3%) |
| **AE related to malaria treatment** | None | 6 (100.0%) | 6 (100.0%) | 6 (100.0%) | 6 (100.0%) | 6 (100.0%) | 6 (100.0%) |
