# Supplementary figures and images for: Controlled human malaria infection by intramuscular and direct venous inoculation of cryopreserved Plasmodium falciparum sporozoites in malaria-naïve volunteers: effect of injection volume and dose on infectivity rates
Source: Malar J. 2015 Aug 7;14:306. doi: 10.1186/s12936-015-0817-x (PMC4527105; doi:10.1186/s12936-015-0817-x)

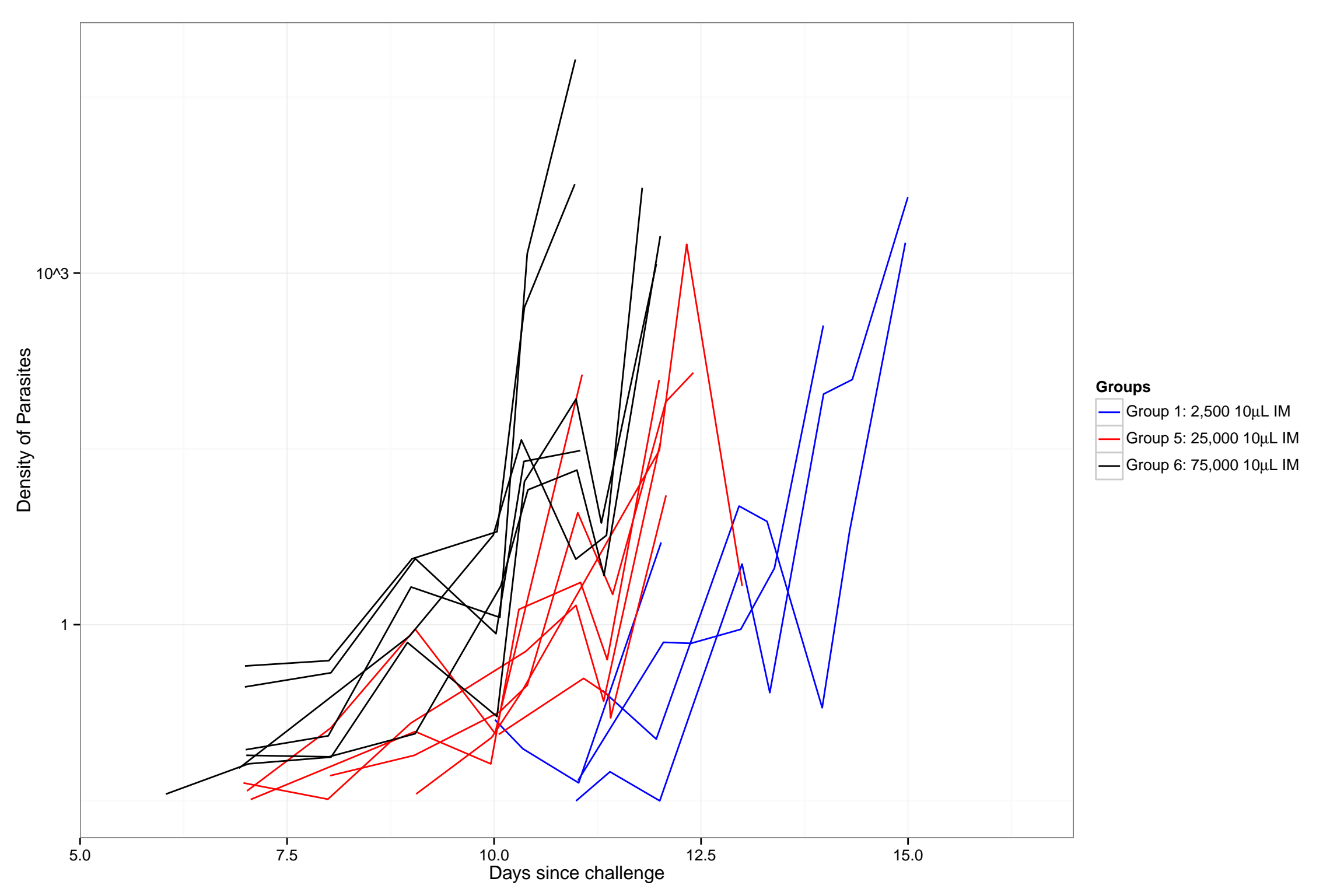

Supplement: Additional file 6: — Parasite kinetics measured by qPCR from Groups 1 (2,500 10μL IM), 5 (25,000 10μL IM) and 6 (75,000 10μL IM). This figure shows parasite densities measured by qPCR, plotted in a single chart and different colors per group, from Groups inoculated by IM injection with increasing doses of 2,500 PfSPZ, 25,000 PfSPZ and 75,000 PfSPZ in a volume of 10μL. The higher the PfSPZ dose inoculated, the sooner sub-microscopic parasite densities are detected. Density units are in parasites/mL. Limit of quantification of qPCR was 30 parasites per mL. [file 12936_2015_817_MOESM6_ESM.pdf]

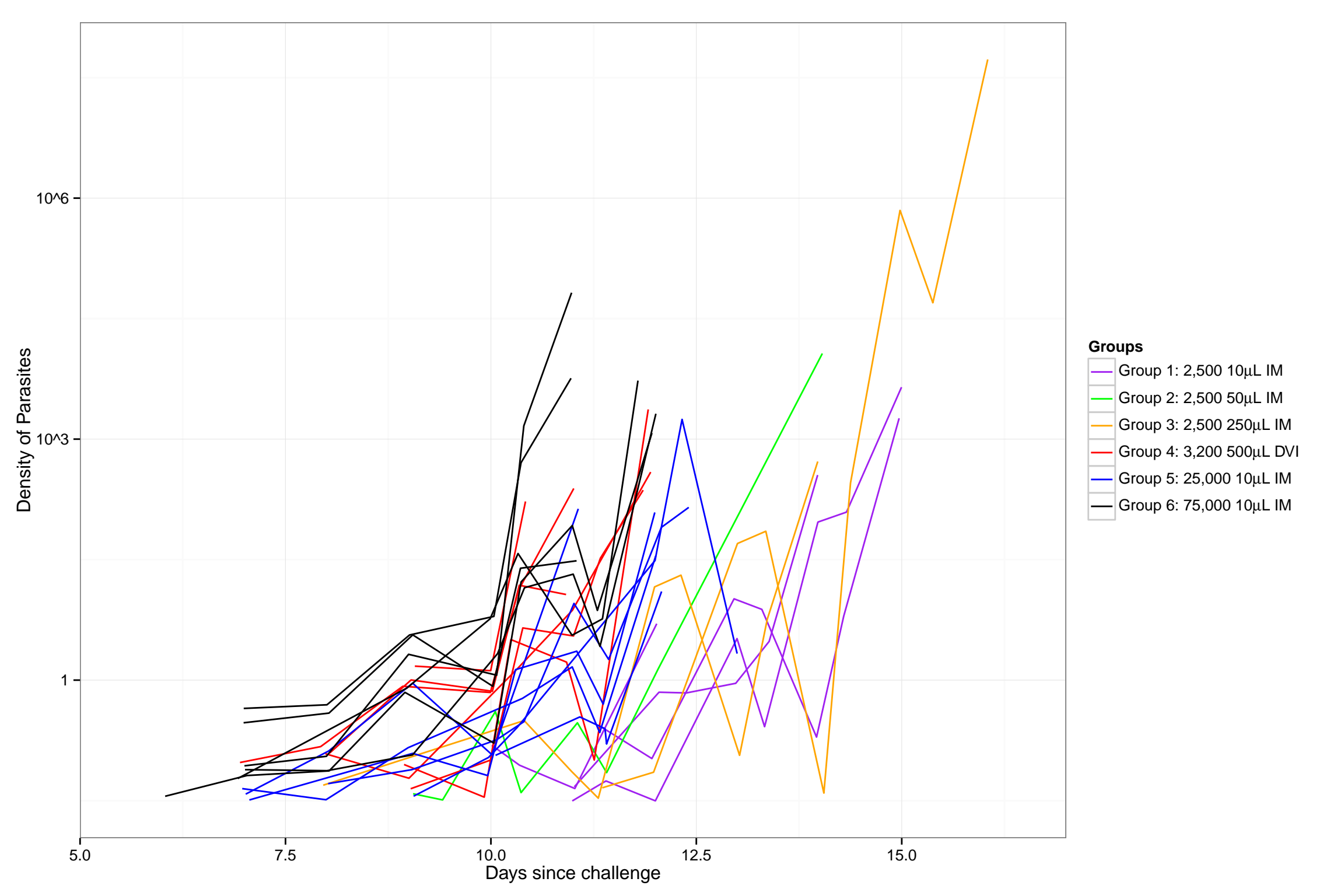

Supplement: Additional file 7: — Parasite kinetics measured by qPCR from all Groups. This figure shows parasite densities measured by qPCR, plotted in a single chart and different colors, from all inoculation Groups. [file 12936_2015_817_MOESM7_ESM.pdf]
