# Supplementary material for: Controlled human malaria infection by intramuscular and direct venous inoculation of cryopreserved Plasmodium falciparum sporozoites in malaria-naïve volunteers: effect of injection volume and dose on infectivity rates
Source: Malar J. 2015 Aug 7;14:306. doi: 10.1186/s12936-015-0817-x (PMC4527105; doi:10.1186/s12936-015-0817-x)
Supplement: Additional file 8: — Pre-patent periods and time to positivity by qPCR from all Groups. This table provides data of the pre-patent periods (days) and time to positivity by qPCR (days) per volunteer from all Groups. [file 12936_2015_817_MOESM8_ESM.docx]

**Additional file 8. Pre-patent periods and time to positivity by qPCR from all groups.**

| **Group** | **Volunteer code** | **Pre-patent period (days)** | **Time to positivity by qPCR (days)** |
| --- | --- | --- | --- |
| Group 1  2,500 PfSPZ 10 µL x 2 IM | BA01-002 | 13.98 | 11.01 |
|  | BA01-006 | 14.97 | 11.31 |
|  | BA01-009 | 15.00 | 11.40 |
|  | BA01-013 | 12.02 | 10.01 |
| Group 2  2,500 PfSPZ 50 µL x 2 IM | BA01-018 | 14.03 | 9.05 |
| Group 3  2,500 PfSPZ 250 µL x 2 IM | BA01-021 | 16.05 | 10.98 |
|  | BA01-033 | 13.98 | 7.96 |
| Group 4  3,200 PfSPZ 500 µL x 1 DVI | BA01-036 | 11.01 | 6.95 |
|  | BA01-046 | 10.42 | 9.07 |
|  | BA01-052 | 12.28 | 7.99 |
|  | BA01-054 | 10.91 | 8.04 |
|  | BA01-065 | 11.85 | 9.02 |
|  | Ba01-043 | 12.20 | 8.94 |
| Group 5  25,000 PfSPZ 10 µL x 2 IM | BA01-037 | 12.38 | 10.06 |
|  | BA01-039 | 12.41 | 8.02 |
|  | BA01-044 | 13.29 | 9.06 |
|  | BA01-051 | 12.35 | 6.97 |
|  | BA01-058 | 11.06 | 7.02 |
|  | BA01-067 | 11.98 | 7.06 |
| Group 6  75,000 PfSPZ 10 µL x 2 IM | BA01-035 | 11.79 | 6.92 |
|  | BA01-040 | 10.98 | 6.99 |
|  | BA01-048 | 10.98 | 7.00 |
|  | BA01-055 | 11.04 | 6.99 |
|  | BA01-063 | 12.01 | 7.01 |
|  | BA01-069 | 11.96 | 6.04 |

Pre-patent period: the time between PfSPZ Challenge injection and first positive thick blood smear; qPCR: quantitative polymerase chain reaction.
